# *Neisseria meningitidis* Induces Brain Microvascular Endothelial Cell Detachment from the Matrix and Cleavage of Occludin: A Role for MMP-8

**DOI:** 10.1371/journal.ppat.1000874

**Published:** 2010-04-29

**Authors:** Alexandra Schubert-Unkmeir, Christian Konrad, Heiko Slanina, Florian Czapek, Sabrina Hebling, Matthias Frosch

**Affiliations:** Institute of Hygiene and Microbiology, University of Würzburg, Würzburg, Germany; Northwestern University Feinberg School of Medicine, United States of America

## Abstract

Disruption of the blood-brain barrier (BBB) is a hallmark event in the pathophysiology of bacterial meningitis. Several inflammatory mediators, such as tumor necrosis factor alpha (TNF-α), nitric oxide and matrix metalloproteinases (MMPs), contribute to this disruption. Here we show that infection of human brain microvascular endothelial cells (HBMEC) with *Neisseria meningitidis* induced an increase of permeability at prolonged time of infection. This was paralleled by an increase in MMP-8 activity in supernatants collected from infected cells. A detailed analysis revealed that MMP-8 was involved in the proteolytic cleavage of the tight junction protein occludin, resulting in its disappearance from the cell periphery and cleavage to a lower-sized 50-kDa protein in infected HBMEC. Abrogation of MMP-8 activity by specific inhibitors as well as transfection with MMP-8 siRNA abolished production of the cleavage fragment and occludin remained attached to the cell periphery. In addition, MMP-8 affected cell adherence to the underlying matrix. A similar temporal relationship was observed for MMP activity and cell detachment. Injury of the HBMEC monolayer suggested the requirement of direct cell contact because no detachment was observed when bacteria were placed above a transwell membrane or when bacterial supernatant was directly added to cells. Inhibition of MMP-8 partially prevented detachment of infected HBMEC and restored BBB permeability. Together, we established that MMP-8 activity plays a crucial role in disassembly of cell junction components and cell adhesion during meningococcal infection.

## Author Summary

A crucial step in the pathogenesis of bacterial meningitis is the disturbance of cerebral microvascular endothelial function, resulting in blood-brain barrier (BBB) breakdown. Matrix metalloproteinases (MMPs) have been implicated in BBB damage in bacterial meningitis in several studies. MMPs are a family of zinc-dependent endopeptidases that catalyze the proteolysis of extracellular matrix proteins, but can also cleave a range of other molecules, including cell adhesion molecules. In this study we showed that brain endothelial cells produced MMPs—in particular MMP-8—upon infection with *Neisseria meningitidis*, a bacterium that causes meningitis and septic shock. We found that MMP-8 was then involved in disruption of the tight junction protein occludin. In addition to the effect of MMP-8 on the tight junction component, MMP-8 activity also accounted for brain endothelial cell detachment that occurred during prolonged time of infection with *N. meningitidis*. When we inhibited MMP-8 activity, occludin disruption was completely abolished and cell detachment could be partially prevented, which resulted in restored BBB permeability. Our data reveal a molecular mechanism of cellular dysfunction during meningococcal meningitis that enhances our understanding how MMPs affect cerebral endothelial function and that can aid in our understanding and prevention of this disease.

## Introduction

Despite improved antimicrobial therapy, bacterial meningitis is still a cause of high mortality and severe neurological morbidity in childhood [1]. *Neisseria meningitidis* is one of the most common causes of bacterial meningitis in Northern Europe and the United States [2], [3]. During meningitis, the host inflammatory response encompasses a variety of detrimental pathophysiological changes, involving increased blood–brain barrier (BBB) permeability, increased CSF outflow resistance, brain edema, elevated intracranial pressure, and alterations in cerebral blood flow [4]. These pathophysiological changes lead to long-term neurological deficits in approximately one-third of the patients [5]–[7].

Several mediators have been shown to affect the BBB permeability. These include reactive oxygen species, nitric oxide, peroxynitrite, matrix metalloproteinases (MMPs), tumour necrosis factor-α (TNFα)-converting enzyme (TACE), transforming growth factor-β1 (TGFβ1), arachidonic acid metabolites, proinflammatory neuropeptides and caspases [8]–[12]. Moreover, experimental and clinical studies suggested that cytokines and chemokines also play an important role in the pathophysiology of BBB disruption during bacterial meningitis. However, the mechanism by which the BBB is damaged during bacterial meningitis is still a matter of debate.

A role of MMPs in BBB damage in bacterial meningitis has been implicated in several studies [13], [14]. In particular MMP-8 and MMP-9 are upregulated in CSF of children with bacterial meningitis, levels being 10 to 1000-fold higher than in viral meningitis [15]. The increase of MMP-8 is a specific feature of bacterial meningitis [14]. MMPs are a family of zinc-dependent endopeptidases that catalyze the proteolysis of a broad spectrum of extracellular matrix (ECM) and basement membrane proteins [16]. MMPs also cleave a range of other molecules, including cytokines, chemokines and growth factors. Neutrophils, glial cells, vascular smooth muscle cells and endothelial cells can produce MMPs upon stimulation. The ability to disrupt the subendothelial basement membrane in cerebral capillary endothelial cells make MMPs likely candidates as effector molecules of BBB breakdown. Intriguingly, MMPs are also implicated in the regulation of cell survival and death [17]. The adherence of cells to the ECM provides survival signals through mechanisms that include the activation of integrin receptors that have engaged particular ECM proteins. When such anchored cells are detached from the substratum, the loss of integrin signaling can result in apoptosis, a phenomenon named anoikis [18], [19].

Alterations of the cerebral microvascular endothelium during bacterial meningitis have been intensively studied. Former experimental studies with *Escherichia coli*, *Streptococcus pneumoniae*, *Haemophilus influenzae* revealed that these bacteria induced functional and morphologic alterations of the BBB [20], which were characterized by an early increase in pinocytotic vesicle formation and a preceding disruption of intercellular tight junctions during time course. On the other hand *in vitro* BBB models have turned out that a number of meningitis-causing pathogens transmigrate across the BBB without barrier disruption [21]–[27]. *N. meningitidis* has been found to transmigrate across T84 epithelial cell monolayers without disruption of tight junctions and sustained electrical resistance [28], [29]. However, only recently type IV pili of *N. meningitidis* have been shown to induce the formation of ectopic early junction-like domains causing the weakening of endothelial cell-cell tight junctions [30].

In this study, we demonstrate that *N. meningitidis* infection results in physiological and morphological alterations of brain endothelial cells after prolonged time of infection. Bacteria induced progressive cell detachment from the matrix and disruption of the tight junction protein occludin. We evaluated MMP-8 activity and the influence of MMP-8 activity on both cell junction components and cell adhesion interaction. We also addressed the question of whether the major virulence factor of meningococci, the polysaccharide capsule, had any influence on cell detachment and/or tight junction proteins.

## Results

### Effect of *Neisseria meningitidis* on endothelial permeability

**Figure 1:**
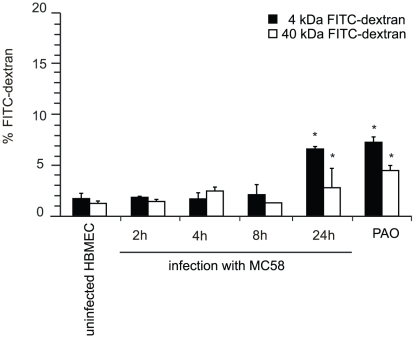
*N. meningitidis* infection alters the paracellular permeability of HBMEC. HBMEC were grown on matrigel–coated 3 µm pore size Transwell filter for 5 days. HBMEC were apically infected with *N. meningitidis* strain MC58 and the paracellular flux of 4–kDa and 40–kDa FITC-dextran was measured at indicated time points. Shown is the total amount of FITC-dextran (in percent) transported from the apical to the basolateral compartment. The graph shows mean values ± SD of three independent experiments done in duplicate. Non-infected cells incubated in phenylarsenoxid (PAO) buffer served as a positive control for tight junction disruption. * *P*<0.05.

To analyze the physiological properties of human brain microvascular endothelial cells (HBMEC) after exposure to *N. meningitidis*, we first evaluated the integrity of polarized endothelial cells by measuring the transendothelial flux of fluorescently labeled dextrans (FITC-dextran). HBMEC were grown onto matrigel-coated Transwell filters for 5 days to obtain confluence. HBMEC monolayers were treated with a well-charaterized encapsulated serogroup B strain (MC58) [31], [32] for indicated times and diffusion of FITC-dextrans with molecular masses of 4 and 40 kDa from the apical to the basal compartment was estimated. About 38% of FITC-dextran was transported in a 30 min period from the apical to the basal compartment when matrigel-coated filters without cells were analyzed, indicating that matrigel coating allowed free diffusion of the tracer (data not shown). Changes in the permeability of HBMEC after exposure to *N. meningitidis* strain MC58 (10^7^ bacteria ml^−1^) were seen. As shown in Figure 1, infection of HBMEC with *N. meningitidis* increased the transport of FITC-dextrans from the apical chamber to the basolateral chamber in a time-dependent manner. After 24 h of infection, the percentage of dextran flux in uninfected cells was 1.73±0.53 and 1.25±0.24 for 4–kDA and 40–kDA FITC-dextran, respectively, whereas *N. meningitidis* significantly increased the dextran flux to 6.62±0.25 and 2.8±0.18 for 4–kDA and 40–kDA FITC-dextran, respectively, at 24 h post-infection (p.i.) (Figure 1). A further increase in FITC-dextran transport across the HBMEC monolayer was seen when 10^8^–10^10^ bacteria per ml were added (data not shown).

### *N. meningitidis infection* induced release of cell-cell contact involving occludin relocation

**Figure 2:**
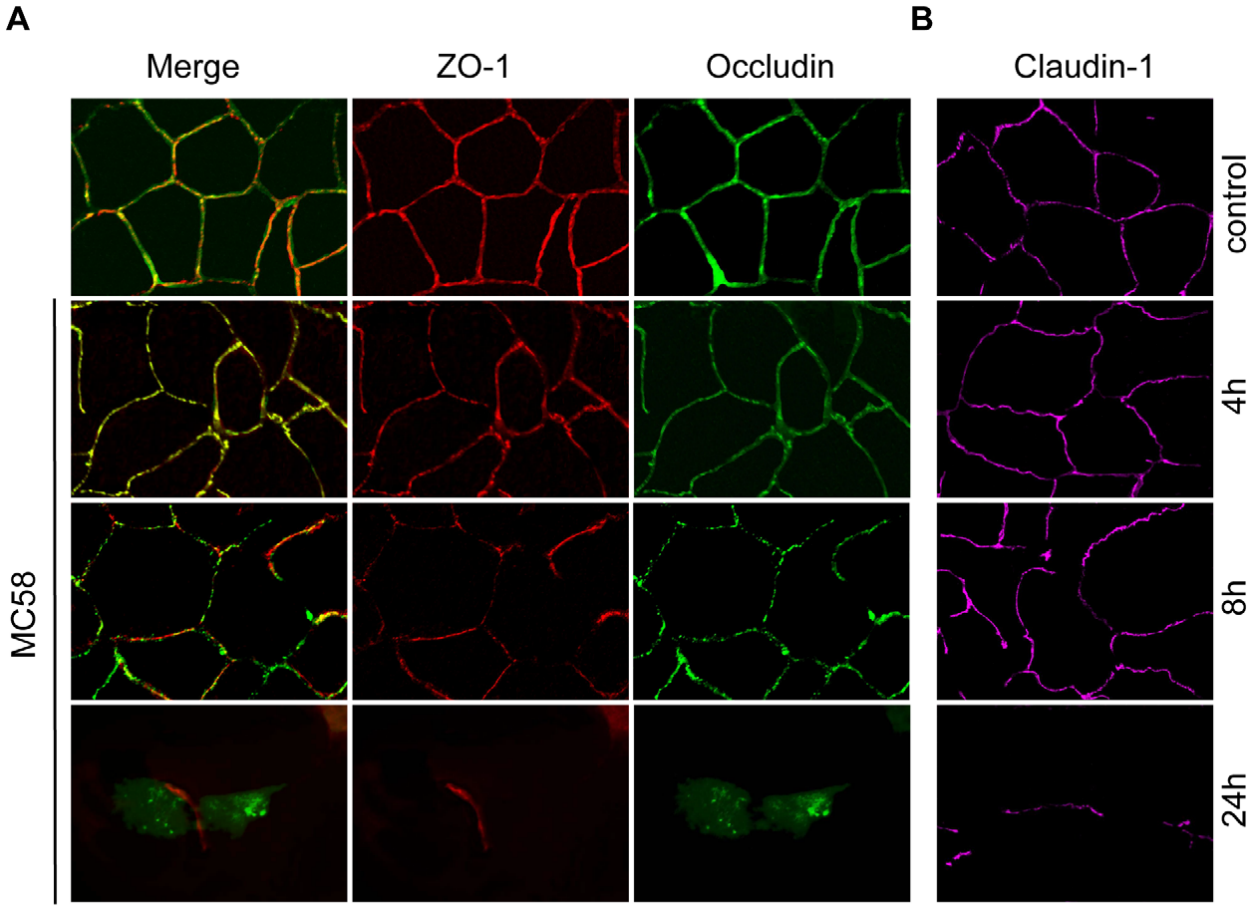
Effect of *N. meningitidis* on the tight junction proteins occludin, ZO-1 and claudin-1. HBMEC were infected with *N. meningitidis* strain MC58 for indicated time points and cells were immunostained for (A) occludin, ZO-1 and (B) separately stained for claudin-1. Control, non-infected HBMEC. Control cells that have been stained for occludin showed the expected localizations. Cells infected with *N. meningitidis* strain MC58 showed dissociation of occludin (green fluorescence) from the cell membrane and a diffuse localization in the cytoplasm at 24 h p.i. Localization of ZO-1 (red fluorescence) and claudin-1 (magenta fluorescence) was not changed in the remaining cells.

Multiple mechanisms may be involved in increased endothelial permeability, such as cell contraction and retraction, enhanced transcellular vesicle transport, disruption of intercellular junctions and cell death. Since the maintenance of the impermeabilty of polarized endothelial cells requires formation of specialized complexes consisting of tight junctions at the apicolateral cell surface [33], we first visualized the localization and expression of the tight junction proteins occludin, claudin-1 and ZO-1 in HBMEC during *N. meningitidis* infection. Non-infected HBMEC showed uniform staining of occludin, claudin-1 and the tight junction-associated cytoplasmatic protein ZO-1 (Figure 2A and B). No differences of the localization of occludin were detected for up to 4 h following bacterial exposure. At 8 h p.i., the distribution of occludin was no longer uniform, and a dotted appearance was observed. When HBMEC were infected with *N. meningitidis* MC58 for a 24-hour period, a complete dissociation of occludin from the membrane was seen. Cells were more irregular in shape and appeared more rounded in interference contrast images (data not shown). We furthermore observed that numerous cells were detached from glass slides or filters at 24 h p.i. during the experimental process of immunfluorescence microscopy.

In contrast to the disruption of occludin localization, ZO-1 and claudin-1 staining was unchanged even in the residual cells at 24 h p.i. (Figure 2A and B).

### *N. meningitidis* infection triggers occludin cleavage

**Figure 3:**
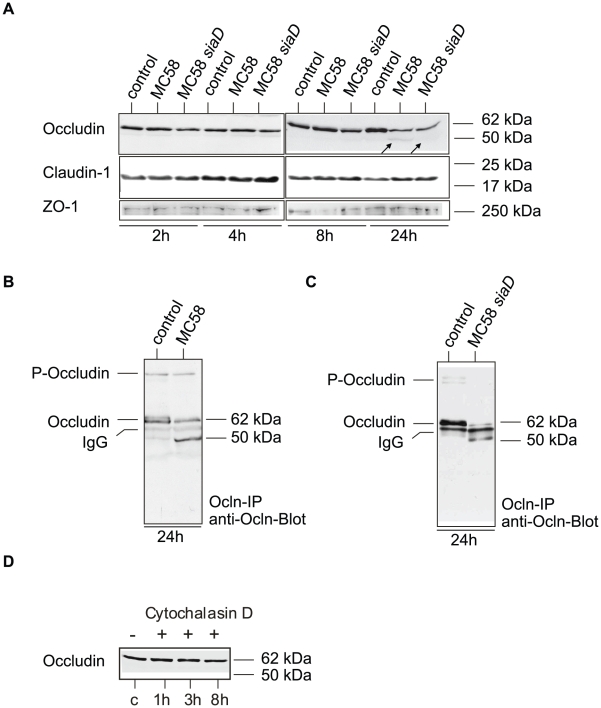
*N. meningitidis* infection of HBMEC causes degradation of occludin. (A) HBMEC cell lysates were analyzed by Western blotting with anti-occludin (clone 19), anti-ZO-1 and anti-claudin-1 after infection with *N. meningitidis* strain MC58 and MC58 *siaD* for 2, 4, 8 and 24 h. A weak specific occludin fragment with an apparent molecular mass of 50–kDa is generated during infection at 24 h p.i. (B and C) Western blot analyses after occludin-immunoprecipitation (ocln-IP), with monoclonal anti-occludin antibody (anti-ocln, clone OC-3F10) showing the generation of the 50-kDa fragment and decrease of the full-length occludin. (D) Detection of occludin by Western blot analysis in Cytochalasin D treated HBMEC lysates.

HBMEC cells were furthermore characterized for tight junction expression by immunoblotting. Cell lysates were generated at different time points after infection with *N. meningitidis* MC58 and analyzed for occludin expression. To further evaluate the role of the major virulence factor of meningococci, the polysaccharide capsule, an isogenic unencapsulated mutant MC58 *siaD* [34] were included in the experiments. While both isolates efficiently adhere to HBMEC, loss of capsule formation resulted in significant increase of meningococcal uptake, which is due to unmasking of outer membrane proteins such as the Opc protein (Figure S1). The Opc protein has recently been shown to be mainly involved in internalization into HBMEC [34]. Infection with both meningococcal strains resulted in generating a weak lower-sized 50-kDa occludin fragment in infected HBMEC at 24 h p.i. (Figure 3A). Whole cell lysates were furthermore subjected to immunoprecipitation: after immunoprecipitation with an occludin-specific monoclonal antibody (clone OC-3F10) recognizing the C-terminal part of occludin, the samples were again analyzed by immunoblotting. As shown in Figures 3B and C the 50-kDa cleavage fragment was clearly visible, while the intensity of the 62-kDa band representing the full-length protein decreased. We next assessed the amounts and presence of claudin-1 and ZO-1 in HBMEC infected with *N. meningitidis* by immunoblot analysis. Immunoblots revealed no changes in the levels of these proteins in infected HBMEC analyzed for a 24-hours time period (Figure 3A). Taken together, these findings indicate that *N. meningitidis* infection induced selective degradation of occludin, which resulted in its dissociation from the tight junctions.

To verify that occludin cleavage was a specific result in *N. meningitidis-*infected cells, HBMEC were treated with Cytochalasin D, which also promotes a round cell shape in endothelial cells, and analyzed for occludin expression by immunoblot analysis. As shown in Figure 3D no cleavage product was observed when HBMEC were treated with Cytochalasin D even at prolonged time of >8 h (data not shown).

### Cleavage of occludin involves MMP-8 activity

**Figure 4:**
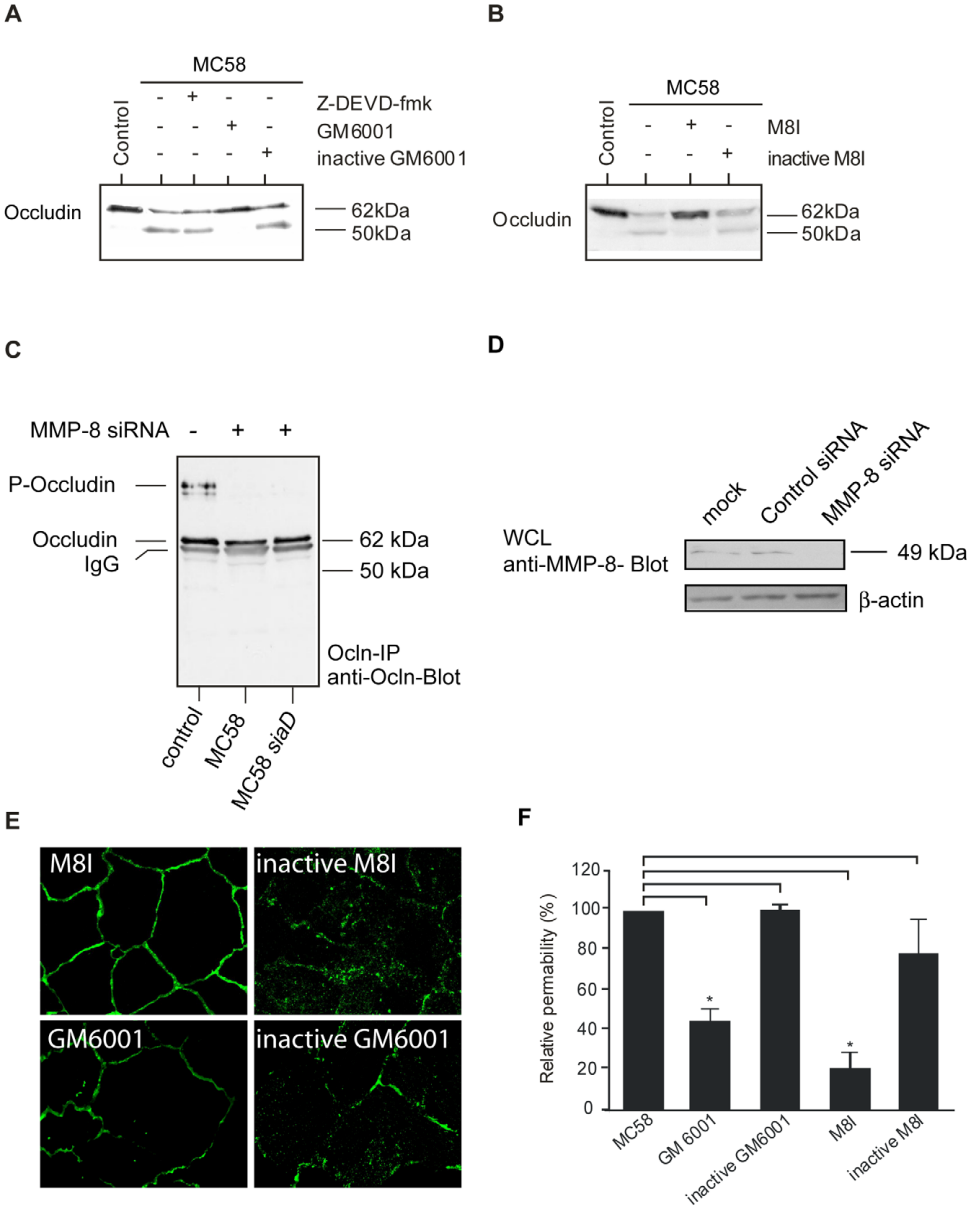
Immunoblot analysis demonstrating that occludin proteolysis after *N. meningitidis* infection is blocked by MMP inhibitors and MMP-8 siRNA transfection. HBMEC were incubated with *N. meningitidis* strain MC58 for a 24-hour period. (A) 60 min prior to bacterial infection, caspase inhibitor Z-DEVD-fmk (50 µM), the broad-spectrum MMP inhibitor GM6001 (20 µM) and the inactive form of GM6001 (20 µM) or (B) a specific MMP-8 inhibitor (M8I) (20 µM) and inactive MMP-8 inhibitor (inactive M8I) (20 µM) were added. Inhibitors were present during the whole time period. Cell lysates were separated on acrylamide gels and transferred to nitrocellulose membranes. Primary anti-occludin mAb (clone OC-3F10) was visualized with IgG alkaline phosphatase-conjugated secondary antibody and enhanced chemiluminescence. Immunoblots are representatives of at least three independent experiments. (C) HBMEC were either transfected with MMP-8 siRNA oligos or control siRNA (see Figure S2). Transfected cells were serum starved for 72 h and then infected with *N. meningitidis* MC58 and MC58 *siaD* or left uninfected. Proteins were lysed, immunoprecipitated (Ocln-IP), and stained with a monoclonal anti-occludin antibody (anti-Ocln) showing the preservation of the 62–kDa full length occludin. (D) Effective silencing of MMP-8 expression in HBMEC transfected with MMP-8 siRNA was shown by immunoblotting with anti-MMP-8 antibody. Antibody against β-actin was used to control the equal amounts of proteins in the lysates. (E) *N. meningitidi*s–induced relocation of occludin was prevented in the presence of the general MMP inhibitor GM6001 and the more specific MMP-8 inhibitor (M8I). (F) Paracellular permeability is regulated by MMPs. Inhibition of MMP activity by GM6001 and a specific MMP-8 inhibitor (M8I) abolished *N. meningitidis*-induced paracellular permeability of 40–kDa FITC-dextran at 24 h p.i. * *P*<0.05.

As occludin contains a putative extracellular matrix metalloproteinase (MMP) cleavage site within the first extracellular loop [35], we hypothesized that the cleavage product was the result of MMP-dependent cleavage. The 50-kDa weight cleavage fragment shown in Figure 3 B and C can be attributed to a cleavage within the first extracellular loop because cleavage at any other site would result in smaller or bigger sized fragments. To verify this hypothesis, HBMEC were infected with *N. meningitidis* strain MC58 in the presence of the broad-spectrum MMP inhibitor GM6001 and cell lysates were again analyzed by immunoblotting. Actually, formation of the 50-kDa fragment was blocked in the presence of the GM6001 (Figure 4A). The inactive form of GM6001 did not prevent occludin proteolysis (Figure 4A). Since GM6001 is a general MMP inhibitor with low-nanomolar inhibition of MMP-1, -2, -3, -8, and -9, we next assessed the involvement of specific MMPs by using the specific MMP-3, MMP-8 and MMP-2/9 inhibitors, MMP-3 inhibitor II, MMP-8 inhibitor I and MMP-2/9 inhibitor I. In the presence of specific MMP-8 I inhibitor formation of the occludin cleavage product was again prevented, whereas the inactive form of MMP-8 did not prevent occludin cleavage (Figure 4B).

A caspase cleavage site in the C-terminal cytoplasmic domain has recently been described [35]. To further exclude the possibility that the 50–kDa occludin fragment was due to apoptotic effects in HBMEC after *N. meningitidis* infection, cells were also pre-incubated with a pan-caspase inhibitor. In the presence of the membrane-permeable caspase inhibitor Z-DEVD-fmk, generation of the 50-kDA fragment was not impaired (Figure 4A).

### Transfection of HBMEC with MMP-8 siRNA blocked occludin cleavage during infection

To support the involvement of MMP-8 in *N. meningitidis*-induced cleavage of occludin, we used RNA-mediated interference to knock down the expression for MMP-8. HBMEC were transfected with 150 nM MMP-8 siRNA or with control siRNA. 72 h post transfection, cells were infected with *N. meningitidis* for 24 h, lysed, immunoprecipitated and analyzed for occludin expression. HBMEC transfected with MMP-8 siRNA demonstrated the fully preserved 62-kDa band representing the full-length protein in infected HBMEC (Figure 4C), while transfection with control siRNA did not affect occludin cleavage (Figure S2). Inhibition of MMP-8 expression by siRNA was monitored from cell lysates from transfected cells and examined by Western blot (Figure 4D). β–actin detection was used to control for integrity of samples and equal protein loading.

### Occludin redistribution and paracellular permeability is regulated by MMP-acticity

To prove whether inhibition of MMP activity could preserve occludin localization at the cell periphery, occludin distribution was visualized in the presence of GM6001 and the specific MMP-8 inhibitor by immunofluorescence microscopy. As shown in Figure 4E, inhibition of MMP-activity partly prevented relocation of occludin in MC58-infected HBMEC.

Finally, analyses of *N. meningitidis*-induced occludin degradation and cell-detachment by means of permeability studies were also suggestive to an involvement of MMPs in this process: Inhibition of MMP activity by GM6001 and the specific MMP-8 inhibitor abolished *N. meningitidis*-induced paracellular permeability of 40 kDa FITC-dextran measured at 24 h p.i. (*P*<0.05) (Figure 4F).

### Secretion of active Matrix metalloproteinases (MMPs)

**Figure 5:**
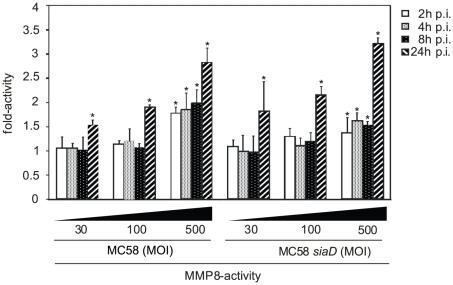
HBMEC were incubated with MC58 and MC58 *siaD* using indicated concentration of bacteria (MOIs of 10, 30 and 500) and matrix metalloproteinase (MMP)-8 activity was measured in supernatants collected from infected HBMEC at 2, 4, 8, and 24 h p.i. using the Sensolyte 520 MMP-8 assay kit.

Since MMP-8 resulted in cleavage of occludin we next analyzed whether infection of HBMEC triggers release of active MMPs. We determined a time-course of MMP-8 activity in the supernatants of infected HBMEC using a 5-FAM/QXL 520 FRET peptide as a substrate. HBMEC were infected with both strains MC58 and MC58 *siaD* using different bacterial concentrations and MMP-8 activity was assayed at 2, 4, 8, and 24 h p.i. As shown in Figure 5, infection of HBMEC with both strains induced a time and dose-dependent secretion of active MMP-8 in the supernatant. Time-course of active MMP secretion paralleled occludin cleavage. Increasing the MOI from 30 to 500 significantly enhanced the release of active MMP-8. Capsule expression did not contribute to active MMP-8 release as no difference between both strains was observed.

### HBMEC detachment during *N. meningitidis* infection

**Figure 6:**
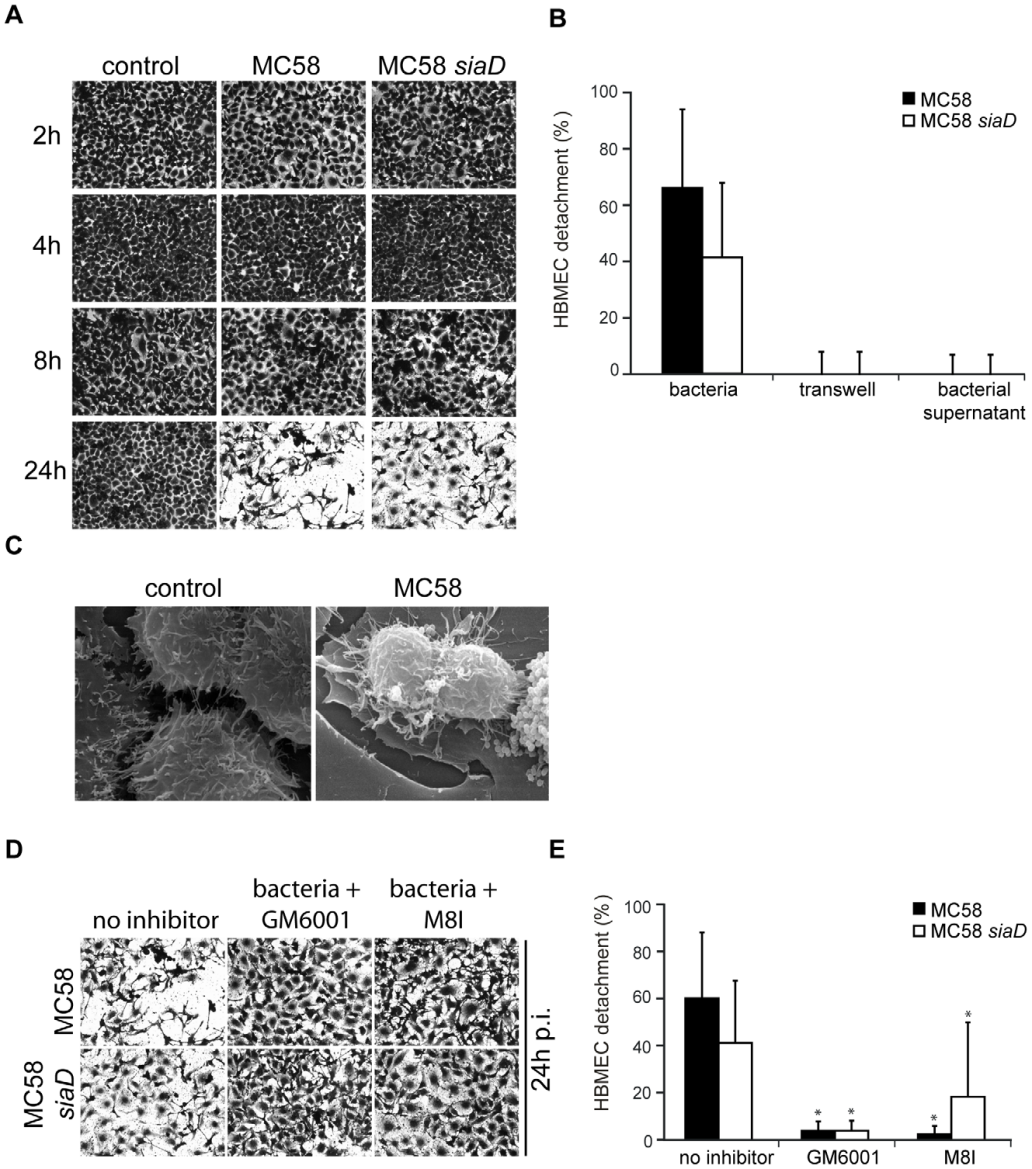
*N. meningitidis* infection triggers time-dependent detachment of HBMEC. (A) Confluent monolayers of HBMEC were infected with *N. meningitidis* MC58 and the unencapsulated isogenic mutant strain MC58 *siaD* for a 24-hour period. Cells were used in detachment assays after fixation and staining with crystal violet (CV). Microscopic photos of the average cell density were taken at ×10 magnification for visualization purposes. (B) Following microscopy, the percentage of detached cells was determined and is expressed as a percentage (mean ± SD of two wells from five independent assays) relative to detachment observed in uninfected control cells. (C) Confluent monolayers of HBMEC were left uninfected (control) or were infected with *N. meningitidis* MC58. Cultures were fixed in situ and were analyzed by scanning EM. **Bacteria-induced cell detachment is blocked by inhibitors of matrix metalloproteinases (MMPs).** (D) HBMEC were infected with indicated bacterial strains for 24 h in the presence of the general MMP inhibitor GM6001 and a specific MMP-8 inhibitor (M8I). Cells were used in a detachment assay and remaining cell were stained with CV and photographed at 24 h. Pictures show representative fields of view. (E) The percentage of detached cells in the presence of MMP inhibitors was determined and is expressed as a percentage relative to detachment observed in uninfected control cells. Error bars represent means ± SD of five independent experiments. * *P*<0.05.

Matrixmetalloproteinase activity can result in the processing of the extracellular matrix (ECM), integrins and other proteins. Since numerous cells were detached from glass slides and/or filters during immunfluorescence analysis we decided to analyze HBMEC adherence to the matrix during *N. meningitidis* infection in detail. HBMEC were infected as described above and cell detachment was either visualized or estimated by crystal violet staining. A progressive detachment of HBMEC during infection was observed. The extent of HBMEC detachment varied according to the time of infection: Detachment from the culture support was visible as soon as 8 h p.i. and clearly visible at 24 h p.i. in response to *N. meningitidis* (Figure 6A). Determination of specific cell detachment revealed that approximately 60% of cells detached at 24 h after infection with the wild-type strain *N. meningitidis* MC58 and approximately 40% of cells after infection with the unencapsulated mutant strain MC58 *siaD*, respectively (Figure 6B), indicating that HBMEC detachment occurred independent from capsule expression.

### HBMEC detachment requires adhesion of bacteria

To determine whether soluble factors released from bacteria could promote HBMEC detachment, bacteria were placed above a transwell membrane and detachment of endothelial cells placed in the lower chamber was assessed. As shown in Figure 6B, HBMEC detachment was not induced unless direct bacteria-cell contact occurred, suggesting that injury of HBMEC monolayers required adhesion of the bacterium to the cell. Moreover, no detachment was observed when bacterial supernatant was collected and directly added to the cell monolayer (Figure 6B).

To investigate the cellular response in more detail, scanning EM was performed on monolayers of HBMEC that were infected with meningococci: In contrast to uninfected cells, cells infected with MC58 or MC58 *siaD* released cell-cell contact and acquired a rounded morphology that was consistent with bacteria-induced cell detachment (Figure 6C).

### Matrix metalloproteinase (MMP) activity is involved in HBMEC detachment

We next considered the possibility that endothelial cell detachment after interaction with *N. meningitidis* might involve matrix-degrading MMPs. HBMEC were infected with *N. meningitidis* strains MC58 and MC58 *siaD* in the presence of the broad-spectrum MMP inhibitor GM6001 and the specific MMP-8 inhibitor and analyzed for cell detachment. As shown in Figures 6D and E, HBMEC detachment could efficiently be inhibited by GM6001 and the specific MMP-8 inhibitor assessed by crystal violet staining and determination of specific cell detachment.

### Monitoring dynamic cell detachment in real-time using impedance technology

To further prove data observed by crystal violet staining, we established an impedance-based real-time cell electronic sensing system (xCELLigence) (Protocol S1) [36]–[38]. First, we determined the optimal concentration for cell proliferation and adhesion and estimated that seeding of 25,000 cells per well was best suited for further experiments (data not shown). Next, 72 h after seeding HBMEC were infected with *N. meningitidis* MC58 and MC58 *siaD* at an MOI of 30 or left uninfected in the presence or absence of MMP inhibitors. Cell index values, corresponding to the intensity of cell adhesion and detachment, were monitored every 15 min using the xCELLigence system for additional 40 h. The increase of the number of attached cells on the electrodes leads to higher Cell index (CI) values, in which the CI represents a dimensionless unit due to the relative change in electrical impedance, while cell detachment will lead to a decreased CI values. As shown in Figure S3, CI values drastically decreased in *N. meningitidis*-infected HBMEC at 24 h p.i.: The CI decreased from 6.24±0.93 and 6.19±0.81 at the beginning of the infection assay (t = 0 h) to 0.72±0.34 and 0.54±0.26 at 24 h p.i. in MC58 and MC58 *siaD*-infected HBMEC, respectively. Addition of MMP-inhibitors significantly decelerated the decrease of CIs in *N. meningitidis*-infected HBMEC corroborating our findings observed by crystal violet staining (1.29±0.47 and 1.57±0.49 in MC58 and MC58 *siaD*-infected HBMEC in the presence of GM6001 and 1.57±0.49 and 1.49±0.32 for MC58 and MC58 *siaD* in the presence of the specific MMP-8 inhibitor (Figures S3 B and C)).

### *N. meningitidis* induces caspase-independent HBMEC detachment

**Figure 7:**
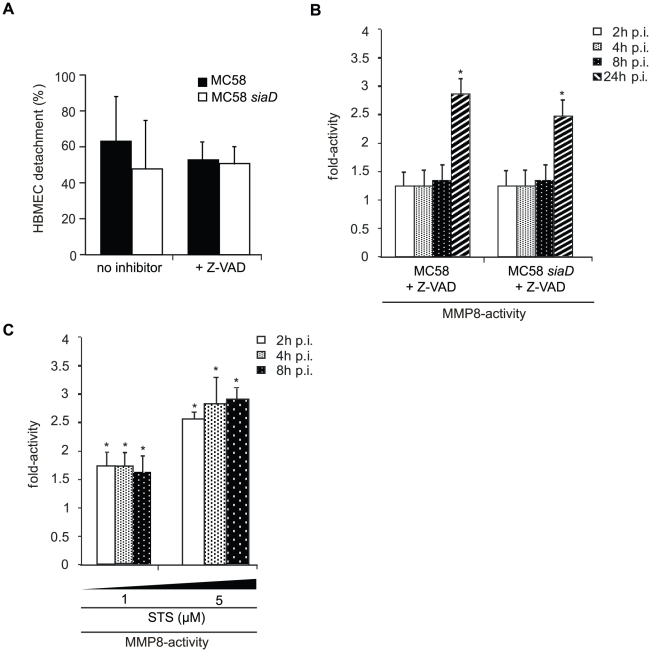
*N. meningitidis* infection triggers caspase-independent detachment of HBMEC. (A) Confluent monolayers of HBMEC were infected with *N. meningitidis* MC58 and the unencapsulated isogenic mutant strain MC58 *siaD* for a 24-hour period in the presence of the pan-caspase inhibitor Z-VAD-fmk. Cells were used in detachment assays and the percentage of detached cells was determined and is expressed as a percentage (mean ± SD of two wells from five independent assays) relative to detachment observed in uninfected control cells. Error bars represent means ± SD of three independent experiments. (B) HBMEC were incubated with MC58 and MC58 *siaD* (MOI 30) and MMP-8 activity was measured in supernatants collected from infected HBMEC at 2, 4, 8, and 24 h p.i. using the Sensolyte 520 MMP-8 assay kit. (C) HBMEC were treated with 1 µM and 5 µM Staurosporin (STS) for 2, 4 and 8 h and MMP-8 activity was measured in the supernatant of STS-treated cells. * *P*<0.05.

Detachment from the matrix is also a late feature of apoptosis in endothelial cells [39]. Pathogenic *Neisseriae* have been shown to induce the expression of apoptosis-related genes and to trigger apoptosis in different cell types [40]–[42]. Yet, caspase-independent detachment of infected cells has recently been shown after infection with the closely related species *N. gonorrhoeae* [43]. To analyze whether caspase activity is required for HBMEC detachment in response to *N. meningitidis*, cells were infected in the presence of the pan-caspase-inhibitor z-VAD-fmk and detachment was measured over a 24-hours time period. Interestingly, *N. meningitidis*-infected HBMEC still continued to detach when Z-VAD-fmk was present in the medium at a concentration of 25 µM (Figure 7).

To determine a mutual dependence from MMP activity of apoptosis signaling, infection assays were carried out in the presence of the pan-caspase inhibitor and MMP-8 activity was estimated in the supernatant. Intriguingly, when infection of HBMEC with *N. meningitidis* MC58 was carried out in the presence of the pan-caspase inhibitor Z-VAD-fmk at a concentration of 25 µM, we still detected an increase in MMP-8 activity during time-period (Figure 7B), indicating that release of active MMP-8 does not necessitate caspase activity. Furthermore, in order to test, whether an apoptotic stimulus was able to induce release of active MMP-8, Staurosporin (STS), a potent apoptosis inductor, was used as control. HBMEC were therefore treated with 1 µM and 5 µM STS for 2, 4 and 8 h and analyzed for active MMP-8 secretion in the supernatant. Indeed, induction of apoptosis by STS resulted in significant release of active MMP-8 already after 2 h post treatment (Figure 7C).

### Rate of apoptosis in adherent and detached HBMEC

Finally, we explored apoptosis rate in adherent and detached (‘floating’) HBMEC after infection with *N. meningitidis* strain MC58. Apoptosis was scored using Annexin-V staining followed by flow cytometry analysis and TdT-mediated dUTP-biotin nick end labeling (TUNEL) staining. Rates of cells in apoptosis are given in Table S1 and Figure S4. While adherent cells displayed low signs of apoptosis, we observed significant apoptosis in cells collected from the supernatant (detached cells, ‘floating’ cells). About 10±2.3% and 54.4±8.6% of detached cells after infection with MC58 and MC58 *sia*D, respectively, at MOI 30 were found to be apoptotic (Table S1).

## Discussion

The present study analyzes the physiological and morphological alterations of endothelial cells comprising the BBB after exposure to *Neisseria meningitidis*. This endothelium differs functionally and morphologically from the endothelial cells of the leaky peripheral vasculature owing to the presence of tight junctions [44]. These cells form the basis of the BBB, which is the primary route penetrated by *N. meningitidis* during meningococcal meningitis [45].

In this study we observed an increase of permeability of brain endothelial cells after prolonged time of infection with *N. meningitidis*. By investigating the mechanism of permeability changes we discovered an unexpected connection between pathogen-induced matrix metalloproteinase (MMP) secretion and disruption of cell-cell connections. Infection with bacteria induced the secretion of active MMPs in the supernatants of infected HBMEC. In particular MMP-8 then participated in the cleavage of the tight junction protein occludin, which resulted in its diffuse accumulation in the cell. Moreover we observed that bacterial adhesion to HBMEC in cell culture resulted in a progressive detachment of the endothelial cell from the underlying matrix. Likewise as observed for occludin disruption MMP activity accounted for cell detachment.

MMPs have already been shown to increase capillary permeability and act as effectors of BBB opening [14]. They target the subendothelial basement membrane ECM as well as cytokines and their receptors [46]. It has only recently been recognized that MMPs can cleave other host proteins, such as cell adhesion molecules – for example, CD44 and αv integrin – as well as some cytokines and tumor necrosis factor (TNF-)α [47] and that they may also play a crucial role in regulation of tight junction dynamics [48], [49]. MMP-7 for example can shed VE-cadherin, a major component of endothelial adherens junctions [50]. MMPs do not have specific cleavage sequences on their target molecules, and cleavage sites are based on the tertiary structure of the protein and not on the primary amino acid sequence. Therefore, it is difficult to predict *a priori* which proteins may be cleaved by each MMP. Occludin contains a putative MMP-cleavage site in the first extracellular loop [35] and cleavage of this loop would result in fragments of about 50-kDa weight as observed in *N. meningitidis*-infected HBMEC. Recent studies have clearly demonstrated that occludin serves as a substrate for MMP-3 and MMP-9 [51], [52]. Furthermore, MMP-7 is involved in disruption of occludin shown in vaginal epithelial cells [53]. However, we are unaware of examples of occludin cleavage involving MMP-8 activity. In this study, we provide several lines of evidence that MMP-8 is involved in occludin disruption. Inhibition of MMP-8 activity using the broad-spectrum inhibitor GM6001 as well as a specific MMP-8 inhibitor reduced *N. meningitidis*-induced occludin cleavage. Furthermore blocking MMP-8 activity by silencing of MMP-8 resulted in inhibition of occludin degradation. In addition, our data showed the involvement of MMP-8 in morphological (occludin relocation) and functional (permeability increase) alterations, and blocking MMP-8 activity preserved occludin attached at the cell membrane under infection and reduced increase of permeability.

The cleavage of occludin mediated by MMP-8 in the first extracellular loop suggested that the C-terminal part of occludin is not affected during infection and therefore remains associated with the membrane and still interacts with ZO-1. This would explain the appearance of ZO-1 in our immunfluorescence microscopy analysis, where ZO-1 localization was not affected during infection and still remained localized to the cell periphery. Otherwise, it has been shown that the function of ZO-1 is not exclusively linked to that of tight junctions [54], but that ZO-1 also interacts with components of adherence junctions such as cadherins. The appearance of ZO-1 at the cell periphery may therefore also be due to binding to adherence junction components. It is therefore an open question, whether the remaining part of occludin stays associated with the apical tight junction or becomes distributed.

Occludin cleavage was observed in response to both meningococcal strains. Western blot analyses suggested that the occludin band in the lane with MC58 *siaD* was lower in intensity compared with MC58. However, densitometric analyses of cleaved occludin and full-length occludin levels taken from three replicates did not show statistically significant differences between both isolates (data not shown). This is in accordance with the observed MMP secretion, since both strains triggered the release of equal amounts of active MMPs.

An interesting study recently published by Coureuil *et al.* [30] gave new insights into the mechanism of BBB transcytosis by *N. meningitidis*. In this work type IV pili of *N. meningitidis* are shown to induce the recruitment of the polarity complex Par3/Par6/PKCζ leading to formation of ectopic junction-like domains at the site of bacterial microcolonies. The formation of the novel junctions weakens the endothelial cell-cell tight junction with opening of the intercellular junctions allowing meningococcal crossing of the BBB by a paracellular route. This cell-cell junction leakage is triggered by type IV pili and occurs at 2 h p.i. The temporal sequence of events suggests that cell-cell junction disruption by rerouting the intercellular junction molecules precedes MMP-8-mediated occludin disruption. However, this initial weakening of tight junctions might favor further protein degradation processes as described for occludin in this study.

In addition to the effect of MMP-8 on tight junction components, adherence of the cell to the basement membrane was also affected in our infection model system. We observed that the contact between the bacteria and HBMEC in culture resulted in the detachment of the endothelial cell from the underlying matrix. We have recently shown that the Opc protein plays a pivotal role in the interaction with brain endothelial cells [34]. Several groups have shown that expression of the polysaccharide capsule, which confers strong protection against the host's immune defense [55], blocks the interaction of meningococci with epithelial and endothelial cells, and only unencapsulated bacteria were able to enter these cell types. It was therefore concluded that the capsule functionally masks membrane proteins, like the Opa and Opc proteins, that mediate a close contact to and the internalisation into host cells. To study the effect of direct contact of *N. meningitidis* via the Opc protein we therefore incorporated an unencapsulated isogenic mutant in our experiments. Our preliminary data on the initiation of cell detachment triggered by *N. meningitidis* revealed that both the encapsulated and unencapsulated mutant strains were capable to promote HBMEC detachment, indicating that the endothelial cell detachment is either not dependent on capsule polysaccharide expression nor on the invasive capacity. We enhanced our end-point assays using an instrument with impedance technology, which allows dynamic monitoring of cell adhesion and detachment during *N. meningitidis* infection. Initial data revealed that 25,000 cells per well were the optimum concentrations for a dynamic monitoring of cell adhesion and detachment. Using this technology real-time analysis of cell detachment nicely correlated with our data observed with the end-point crystal violet staining assays.

Detachment paralleled MMP secretion and was inhibited by the general MMP inhibitor GM6001 and a specific MMP-8 inhibitor. HBMEC detachment might occur due to cleavage of protein components of the ECM by MMPs, resulting in inappropriate ECM interaction. Alternatively, dissociation of transmembrane matrix receptors from the cytoskeleton anchoring proteins might contribute to the phenomenon.

The adherence of cells to the ECM provides survival signals through mechanisms that include the activation of integrin receptors that have engaged particular ECM proteins. When such anchored cells are detached from the substratum the loss of integrin signaling can result in apoptosis, a phenomenon named anoikis [18], [19]. Indeed, detached HBMEC displayed significant signs of apoptosis. On the other hand, endothelial cell detachment has been demonstrated as a late feature of endothelial cells undergoing apoptosis [56] and pathogenic *Neisseriae* have been shown to induce the expression of apoptosis-related genes and to trigger apoptosis in different cell types [40]–[42], [57]. Caspase 3 has been shown to act as an effector for the cytoskeletal remodelling involved in cell “rounding” that occurs before the apoptotic cell detaches [39]. However, we could show that HBMEC still continued to detach in the presence of the pan-caspase inhibitor Z-VAD-fmk, indicating caspase-independend cell detachment. These findings are in line with a recently published study by Kepp and co-workers, who demonstrated that the closely related species *N. gonorrhoeae* triggers a caspase-independent detachment of infected epithelial cells [43]. Moreover, we observed a low rate of apoptosis in remaining adherent cell after infection with *N. meningitidis* strain MC58. The inability of strain MC58 to induce apoptosis has recently been reported by Deghmane *et al.* [42] for HecIB cells.

MMPs have been described to exert cytotoxic effects [17], [58]. Apoptosis can be affected by direct proteolysis of death-inducing signaling components: MMP-7 for example has been described to mediate apoptosis through the generation of a soluble form of Fas ligand that initiates Fas-dependent apoptosis [59]. However, whether both enzyme families are separately activated and impact on cell detachment or cause themselves mutually remains to be elucidated.

Taken together our data support the central role of MMP-8 in the disassembly of both host cell junction components and cell adhesion to the ECM as a consequence of meningococcal infections. The mechanisms by which MMP-8 is activated during meningococcal infection will be one focus of further investigations, as delineating this process will be fundamental to increasing our understanding of the interaction between this major meningitis causing pathogen and brain endothelial cells. However, so far our data suggest that adhesion contributes to detachment of infected cells. This could occur through increasing local delivery of bacterial factors, such as the lipooligosaccharid (LOS). The importance of endotoxin of Gram negative bacteria in mediating cell damage has been documented [60], [61]. Analyzing the specific effect of meningococcal LOS on HBMEC detachment as well as exploring the influence of *N. meningitidis* on further cell junction components will be will be an ambitious task for further investigations.

## Materials and Methods

### Reagents and antibodies

Goat anti-actin (I19) antibody and rabbit anti-ZO-1 (clone I19) were purchased from Santa Cruz Biotechnology (Santa Cruz, CA, USA, 1∶100). Mouse anti-occludin (clone OC-3F10; against C-terminus) was from Zymed (Invitrogen, Ca, USA). Secondary antibodies goat anti-mouse IgG alkaline phosphatase-conjugated antibody and goat anti-rabbit IgG alkaline phosphatase-conjugated antibody were obtained from Invitrogen (Invitrogen, Ca, USA). Alexa Fluor546-conjungated goat anti-rabbit, Alexa Fluor488-conjugated goat anti-mouse antibodies and 4′,6-diamidino-2-phenylindole, dihydrochloride (DAPI) were obtained from Invitrogen. The broad spectrum matrix-metalloproteinase (MMP) inhibitors GM6001 (and its inactive form), the specific MMP inhibitors (MMP-2/MMP-9 Inhibitor I, MMP-8 Inhibitor I, MMP-3 Inhibitor II), as well as the caspase inhibitor Z-D(OMe)E(OMe)VD(OMe)-FMK (Z-DEVD-fmk) and the pan-caspase inhibitor Z-VAD fmk were obtained from Calbiochem (Schwalbach, Germany). Staurosporine (STS) were purchased from Sigma Aldrich (Taufkirchen, Germany). FITC-Annexin V kit was from BioCat (Heidelberg, Germany).

### Bacterial strains, cell culture and infection assays

*Neisseria meningitidis* strain MC58, a serogroup B isolate (United Kingdom, 1983) of the ST-32 complex characterized as serotype B:15:P1.7,16 was kindly provided by E. R. Moxon. Non-encapsulated mutant MC58 *siaD*, used in this study was previously described in detail [34]. The simian virus 40 large T antigen-transformed human brain microvascular endothelial cells (HBMEC) were kindly provided by K. S. Kim [22] and were cultured as previously described [34]. Cells between the 10^th^ and 25^th^ passages were used for infection assays. HBMEC were cultured in T25 flasks (Corning Costar Corporation, Cambridge, MA, USA) to a confluent monolayer. At 48 hours prior to infection, HBMEC were split and seeded on matrigel (BD Matrigel Matrix, Heidelberg, Germany)–coated 24–well tissue culture plates (Sarstedt; Germany) or on matrigel–coated Transwell cell culture chambers (polycarbonate filters, 3.0-µm pore size; Corning Costar Corporation, Cambride, MA, USA) at a density of 5×10^4^ cells per well. Cells were grown to approximately 1×10^5^ cells prior to infection. Monolayers of HBMEC were infected with bacteria at an MOI of 30 unless indicated otherwise for a 24-hour time period. Infections were carried out in the presence of 10% human serum (HS) supplemented RPMI medium. HS were derived from a serum pool (voluntary staff) and heat-inactivated for 30 min at 56°C. Use of HS–supplemented RPMI medium is based on the observation that meningococcal entry is supported by binding of the Opc protein via fibronectin to integrins on HBMEC [34]. Adhesion assays were performed as described previously [34]. We repeatedly tested the wild-type strain and the isogenic capsule deficient mutant for pili, Opa and Opc expression before application to infection assays and after re-isolation from the cell culture using Western blot analysis to exclude variation in the expression level of these meningococcal components. To compare the effects of soluble factors, cells were cultured in transwells with bacteria placed in the upper chamber, separated by a permeable filter (0.4 µm pore size).

### *In vitro* permeability assays

Paracellular permeability was studied by measuring the apical-to-basolateral flux of Fluorescein isothiocyanate (FITC)-dextran (Sigma, St Louis, MO, USA) through confluent HBMEC monolayers. HBMEC were seeded onto matrigel-coated Transwell filters at 5×10^4^ cells/filter in 200 µl of HS–supplemented RPMI medium. The lower compartment was filled with 800 µl of the same medium. HBMEC were grown for 5 days to obtain confluence. About 1.7% of 4–kDa FITC-dextran and 1.1% of 40–kDA FITC-dextran was found in the lower chamber when flux was analyzed in a 30 min period. Matrigel was used in a concentration of 10 µg ml^−1^ which allowed free diffusion of tracers and bacteria (data not shown). To measure paracellular flux, 4–kDa or 40–kDa FITC-dextrans were dissolved in P buffer [10 mM 4-(2-hydroxyethyl)-1-piperazine-ethanesulphonic acid (HEPES), pH 7.4; 1 mM sodium pyruvate; 10 mM glucose; 3 mM CaCl_2_, 145 mM NaCl]. Bacteria were grown as described above, resuspended in HS–supplemented RPMI medium and inoculated on the apical surface of the cell layer at a MOI of 30 unless indicated otherwise. After indicated time points of infection, transwell inserts were replaced to measure paracellular flux. Cells were allowed to equilibrate in P buffer for 20 min and FITC-dextrans were added to the apical compartment to give a final concentration of 1 mg ml^−1^. After 30 min, the basolateral medium was collected and the concentrations of FITC-dextrans were measured with a fluorometer in the presence or absence of inhibitors (excitation 485 nm, emission 535 nm). Non-infected cells served as a negative control while non-infected cells incubated in phenylarsenoxid (PAO/DMSO) buffer served as a positive control for tight junction disruption as described previously [48].

### Electron microscopy

For scanning electron microscopy, cells were fixed in situ with 2% glutaraldehyde/3% formaldehyde buffered in 0.1 M cacodylate, 0.09 M sucrose, 0.01 M CaCl_2_, and 0.01 M MgCl_2_ (pH 6,9) for at least 1 h at 4 C. Samples were dehydrated in a graded series of acetone on ice. After critical point drying from liquid CO_2_, samples were sputter coated with gold/palladium and examined at 15 kV of accelerating voltage in a field emission scanning electron microscope (model DSM962, Zeiss, Oberkochen, Germany). Images were digitally recorded and processed in Adobe Photoshop CS.

### Detachment assay

HBMEC were infected with bacteria (MOI 30) as described. After infection, cells were gently washed with 1xPBS while shaking for 10 min to remove loosely attached cells. Adherent cells were fixed, dyed with crystal violet (0,75% crystal violet, 50% ethanol, 0,25% NaCl, 1,75% formaldehyde) and incubated at room temperature for 30 min. Cells were washed twice with PBS and air dried. Cells were examined with a Zeiss Axiovert 40 CFL inverted light microscope using high NA 10X objective and microscopic photos were taken using the Zeiss AxioCam ICc1 digital camera system. After microscopy, cells were lysed in elution solution (1% SDS in 1xPBS) overnight. The staining intensity was measured in a microplate reader (Tecan) at 620 nm. Bacteria were furthermore separated from the cell monolayer by a transwell filter system (Transwell cell culture chambers, polycarbonate filters, 0.4-µm pore size; Corning Costar Corporation, Cambride, MA, USA) or bacteria were grown to midlog phase, filtered and supernatants were added directly to the cell monolayer. The percentage of detached cells is expressed as a percentage (mean ± SD of two wells from five independent assays) relative to detachment observed in uninfected control cells.

### Immunodetection of occludin, ZO-1 and claudin-1 in infected cultures

HBMEC were grown to confluence on matrigel–coated glass coverslips or on matrigel–coated Transwell filters for localization studies. Monolayers were infected with *N. meningitidis* strains MC58 and MC58 *siaD* (MOI of 30) for 2, 4, 6, 8 and 24 h in HS–supplemented RPMI in the presence or absence of inhibitors. Cells were rinsed gently three times with PBS to remove extracellular non–adherent bacteria and fixed in PBS with 3.7% paraformaldehyde for 20 min at RT. Cells were then rinsed, permeabilized with 0.1% Trition-X-100 in PBS for 15 min and blocked in 1% BSA in PBS for further 45 min. Monolayers were incubated with primary antibodies overnight at 4°C. Primary antibodies were used at the following dilutions: rabbit anti-ZO-1 (1∶50), mouse anti-occludin (clone 19) (1∶500), and mouse anti-occludin (clone OC-3F10) (1∶150). Following incubation with the first antibodies monolayers were washed three times with PBS and than incubated with the appropriate secondary antibodies Alexa 546-conjugated goat anti-rabbit (1∶200) and Alexa 488-conjugated goat anti-mouse (1∶200). Monolayers were viewed on a Zeiss Axio Imager.Z1 microscope equipped with ApoTome. Images were photographed using AxioCam digital Camera and AxioVision software and imported into Adobe Photoshop CS for manuscript preparation.

### Cell lysis and Western blotting

HBMEC monolayers were incubated with *N. meningitidis* strains MC58 and MC58 *siaD* for indicated time points and then washed three times in ice-cold PBS. Proteins were extracted using ice-cold buffer containing 1% Triton-X-100, 20 mM Tris-HCl, 150 mM NaCl, 0.1% SDS, 1% deoxycholic acid, 5 mM EDTA, 1 mM Na_3_VO_4_, 2 mM phenylmethylsulphoyl fluoride (PMSF), 50 µg ml^−1^ pepstatin, 50 µg ml^−1^ chymostatin and 50 µg ml^−1^ antipain. Extracts were spun at 14.000×g for 10 min and the supernatant was removed. β–actin detection was used to control for integrity of samples and equal protein loading. MMP inhibitor GM6001 and the inactive form of GM6001 (20 µM), as well as the specific MMP-8 inhibitor (20 µM) and the inactive form of the MMP-8 inhibitor (20 µM) were added 60 min prior to infection, Z-DEVD-fmk (50 µM) was added 1 h prior to infection. All inhibitors were again added at 6 h p.i.

### SDS-Page and immunoblot analysis

Protein (30–50 µg) from total cell lysates was used for immunoblot analysis. SDS-PAGE (6% acrylamide for ZO-1 and 10–12% acrylamide for occludin and claudin-1) was performed as described previously [62] using Mini-Protean electrophoresis System (Bio-Rad, München, Deutschland). Proteins were transferred electrophoretically on nitrocellulose membranes (Millipore, Bedford, MA) using 30 mA for 1 h. The membranes were incubated overnight at 4°C in blocking buffer (Tris-buffered saline, 0.1% Tween 20 (TBST), 5% skim milk) and than incubated with primary antibodies diluted in blocking buffer for 1 h at RT (rabbit anti-ZO-1 (1∶80), mouse anti-occludin (clone 19) (1∶50), mouse anti-occludin (clone OC-3F10) (1∶2000)). After washing in TBST, the membranes were incubated with the appropriate secondary antibody (goat anti-mouse IgG alkaline phosphatase-conjugated antibody: 1∶10000; goat anti-rabbit IgG alkaline phosphatase-conjugated antibody: 1∶5000) diluted in blocking buffer for 1 h at RT. After washing in TBST, the bands were detected using an enhanced chemiluminescence kit (Amersham), according to the manufactures' instructions. All Western blots are representative of at least three experiments carried out.

### Immunoprecipitation

3 µg monoclonal anti-occludin antibody/sample were added to cleared lysates containing equivalent amounts of protein and incubated for 4 h at 4°C. After addition of protein A/G plus agarose (Santa Cruz Biotechnology, Santa Cruz, CA) and 1 h incubation at 4°C, samples were washed twice with RIPA buffer and twice with Triton buffer (25 mM Hepes (pH 7.4), 1% Triton X-100, 150 mM NaCl, 20 mM MgCl_2_, 10% glycerol, 10 mM sodium pyrophosphate, 100 mM NaF, 1 mM Na_3_VO_4_, and 10 µg ml^−1^ of each aprotinin, leupeptin, and pepstatin). For Western blot analysis, the precipitates were taken up in reducing 2x SDS sample buffer and analyzed as described above.

### siRNA transfection

siRNA (MMP-8 and Control) was synthesized by Santa Cruz Biotechnology. MMP-8 siRNA (sc-35949) is a pool of three target-specific 20–25 base sequences siRNAs designed to knock down expression. Control siRNA (sc-37007) is a non-targeting 20–25 nt siRNA, which consists of a scrambled sequence that will not lead to the specific degradation of any known cellular mRNA. At 75% confluency, HBMEC were mock transfected on 24-well plates or transfected with either 150 nM MMP-8 siRNA or 150 nM control siRNA using 3 µl of HiPerfect Transfection reagent (Qiagen) according to the manufacture's guidelines. MMP-8 expression was monitored 72 h post transfection by Western blot analysis with an anti-MMP-8 antibody (Acris, Herford, Germany). 72 h after transfection cells were infected with bacteria for 24 h before cell lysate collection and immunoprecipitation. Blots were reprobed with a goat anti-β-actin antibody to control for protein loading.

### Fluorometric MMP activity assay

MMP activity was determined using the Sensolyte 520 MMP-8 Assay Kit (AnaSpec, San Jose, CA, USA) following the manufacturer's instructions. Briefly, media from MC58 and MC58 *siaD*-infected HBMEC and non-infected HBMEC were collected 2, 4, 8, and 24 h p.i. and 50 µl of these culture supernatant were incubated with the FAM/QXL 520 fluorescence resonance energy transfer substrate for 1 h in a black 96-well plate at room temperature in the dark. Measurements were made using a Tecan microplate reader (excitation at 360 nm, emission at 465 nm).

### Statistical analysis

Statistical analyses were performed using Student's *t* test, and data were considered significant if *P* was <0.05.

## Supporting Information

Protocol S1 Supplemental Material and Methods and Figure Legends
(0.03 MB DOC)

Figure S1 Time-dependent adherence to and invasion of *N. meningitidis* strain MC58 the unencapsulated isogenic mutant MC58 *siaD* into HBMEC. Confluent monolayers of HBMEC were infected with *N. meningitidis* MC58 and the unencapsulated isogenic mutant strain MC58 *siaD* for a 24-hour period in the presence of 10% HS and either (A) adherence to and (B) uptake by HBMEC was estimated at indicated time points. Error bars represent means ± SD of three independent experiments done in duplicate.
(1.36 MB EPS)

Figure S2 Immunoblot analysis of infected HBMEC transfected with control siRNA. HBMEC were transfected with 150 nM control siRNA. Transfected cells were serum starved for 72 h and then infected with *N. meningitidis* MC58 or left uninfected. Proteins were lysed, immunoprecipitated (Ocln-IP), and stained with a monoclonal anti-occludin antibody (anti-Ocln) showing the occludin cleavage fragment as observed for non-transfected infected HBMEC.
(0.93 MB EPS)

Figure S3 Dynamic monitoring of cell adhesion and detachment of HBMEC after infection with *N. meningitidis*. HBMEC were seeded in 96X microtiter plates (E-Plates) at a density of 25,000 cells per well. The adhesion of the cells were continuously monitored every 15 min for 5 days using the xCELLigence system. Approximately 72 h after seeding, cells were infected with *N. meningitidis* MC58 and MC58 *siaD* in the presence or absence of MMP inhibitors. Adhesion and detachment of cells were continuously monitored every 15 min using the xCELLigence system. At 24 h p.i. cell index (CI) drastically decreased in *N. meningitidis*-infected HBMEC. Addition of MMP-inhibitors significantly decelerated the decrease of CI under infection. CI represents a dimensionless unit due to the relative change in electrical impedance. The CI values of (A) control cell in the presence or absence of MMP-inhibitors, (B) HBMEC infected with MC58 *siaD* and (C) HBMEC infected with MC58 in the presence or absence of MMP-inhibitors are plotted on the graph. Relative values of three independent experiments done in duplicate are shown. * *P*<0.05.
(1.32 MB EPS)

Figure S4 Detection of apoptosis by TUNEL staining. HBMEC were cultured on cover slides and infected with *N. meningitidis* MC58 and MC58 *siaD* for a 24 h time-period. Positive terminal deoxynucleotidyl transferase-mediated dUTP nick end labeling (TUNEL) staining was observed in cells collected from the supernatant (‘floating’ cells), while adherent cell showed rare positive apoptotic cells. Shown are representative photomicrographs from three independent experiments.
(2.42 MB TIF)

Table S1 Evaluation of the rate of apoptosis in HBMEC after infection with *N. meningitidis*. Confluent HBMEC monolayers were infected with different MOIs of *N. meningitidis* MC58 and MC58 *siaD*. After 24 h of incubation adherent and floating cells were separately collected, washed and stained with PI-Annexin V and analyzed by flow cytometry. As a positive control for apoptosis, cells were treated with 1 µM and 5 µM Staurosporine (STS). Data present the mean ± SD of three independent experiments done in duplicate.
(0.03 MB DOC)
