# Supplementary material for: Neisseria meningitidis Induces Brain Microvascular Endothelial Cell Detachment from the Matrix and Cleavage of Occludin: A Role for MMP-8
Source: PLoS Pathog. 2010 Apr 29;6(4):e1000874. doi: 10.1371/journal.ppat.1000874 (PMC2861698; doi:10.1371/journal.ppat.1000874)
Supplement: Protocol S1 — Supplemental Material and Methods and Figure Legends (0.03 MB DOC) [file ppat.1000874.s001.doc]

**Supporting Information**

**Supplemental Material and Methods**

**Monitoring dynamic cell adhesion and detachment in real-time using impedance technology.**

The xCELLigence sytem was used according to the instruction of the supplier (Roche Applied Science and ACEA Biosciences, [62]). HBMEC were seeded in standard microtiter plates (96X microtiter plates; E-Plates (Roche Applied Science) that contain microelectronic sensor arrays. For detachment analyses HBMEC were seeded at a density of 25,000 cells per well and either left uninfected or infected with *N. meningitidis* MC58 and MC58 *siaD* at an MOI of 30 in the presence or absence of MMP inhibitors. Dynamic cell proliferation and intensity of adhesion was monitored every 30 min using the xCELLigence system. Approximately 72 h after seeding, cells were infected with bacteria and cell adhesion and detachment was monitored for further 40 h. Cell-sensor impedance was expressed as an arbitrary unit called the cell index according to the manufacturer’s instructions. The cell index (CI) at each time point is defined as (R_n_-R_b_)/15, where R_n_ is the cell-electrode impedance of the well when it contains cells and R_b_ is the background impedance with media alone. CI values were calculated and plotted on the graph. Standard deviations of duplicates of wells were analyzed with the RTCA Software.

**Analysis and quantitative measurement of apoptosis**

**Annexin V assay.**

To quantify the amount of cellular apoptosis in infected HBMEC, Annexin V staining was performed using the Annexin V- PE apoptosis detection kit (Biovision) according to the manufacturer's specification. Remaining adherent and floating cells were separately collected and stained. The fraction of cells with positive Annexin V staining was defined as cells undergoing apoptosis. All samples were analyzed by flow cytometry on the FACSCalibur flow cytometer (Becton-Dickinson).

**TUNEL assay.**

Apoptosis in *N. meningitidis* infected HBMEC was assessed using the R&D Apoptosis Detection Kit, according to the manufacturer instructions (R&D Systems, Wiesbaden-Nordenstadt, Germany).

**Figure S1:**

**Time-dependent adherence to and invasion of *N. meningitidis* strain MC58 the unencapsulated isogenic mutant MC58 *siaD* into HBMEC.**

Confluent monolayers of HBMEC were infected with *N. meningitidis* MC58 and the unencapsulated isogenic mutant strain MC58 *siaD* for a 24-hour period in the presence of 10% HS and either (A) adherence to and (B) uptake by HBMEC was estimated at indicated time points. Error bars represent means ± SD of three independent experiments done in duplicate.

**Figure S2:**

**Immunoblot analysis of infected HBMEC transfected with control siRNA.**

HBMEC were transfected with 150nM control siRNA. Transfected cells were serum starved for 72 h and then infected with *N. meningitidis* MC58 or left uninfected. Proteins were lysed, immunoprecipitated (Ocln-IP), and stained with a monoclonal anti-occludin antibody (anti-Ocln) showing the occludin cleavage fragment as observed for non-transfected infected HBMEC.

**Figure S3:**

**Dynamic monitoring of cell adhesion and detachment of HBMEC after infection with *N. meningitidis.***

HBMEC were seeded in 96X microtiter plates (E-Plates) at a density of 25,000 cells per well. The adhesion of the cells were continuously monitored every 15 min for 5 days using the xCELLigence system. Approximately 72 h after seeding, cells were infected with *N. meningitidis* MC58 and MC58 *siaD* in the presence or absence of MMP inhibitors. Adhesion and detachment of cells were continuously monitored every 15 min using the xCELLigence system. At 24 h p.i. cell index (CI) drastically decreased in *N. meningitidis*-infected HBMEC. Addition of MMP-inhibitors significantly decelerated the decrease of CI under infection. CI represents a dimensionless unit due to the relative change in electrical impedance. The CI values of (A) control cell in the presence or absence of MMP-inhibitors, (B) HBMEC infected with MC58 *siaD* and (C) HBMEC infected with MC58 in the presence or absence of MMP-inhibitors are plotted on the graph. Relative values of three independent experiments done in duplicate are shown. * *P* < 0.05.

**Figure S4:**

**Detection of apoptosis by TUNEL staining.**

HBMEC were cultured on cover slides and infected with *N. meningitidis* MC58 and MC58 *siaD* for a 24 h time-period. Positive terminal deoxynucleotidyl transferase–mediated dUTP nick end labeling (TUNEL) staining was observed in cells collected from the supernatant (‘floating’ cells), while adherent cell showed rare positive apoptotic cells.

Shown are representative photomicrographs from three independent experiments.
