# Supplementary figures and images for: Neisseria meningitidis Induces Brain Microvascular Endothelial Cell Detachment from the Matrix and Cleavage of Occludin: A Role for MMP-8
Source: PLoS Pathog. 2010 Apr 29;6(4):e1000874. doi: 10.1371/journal.ppat.1000874 (PMC2861698; doi:10.1371/journal.ppat.1000874)

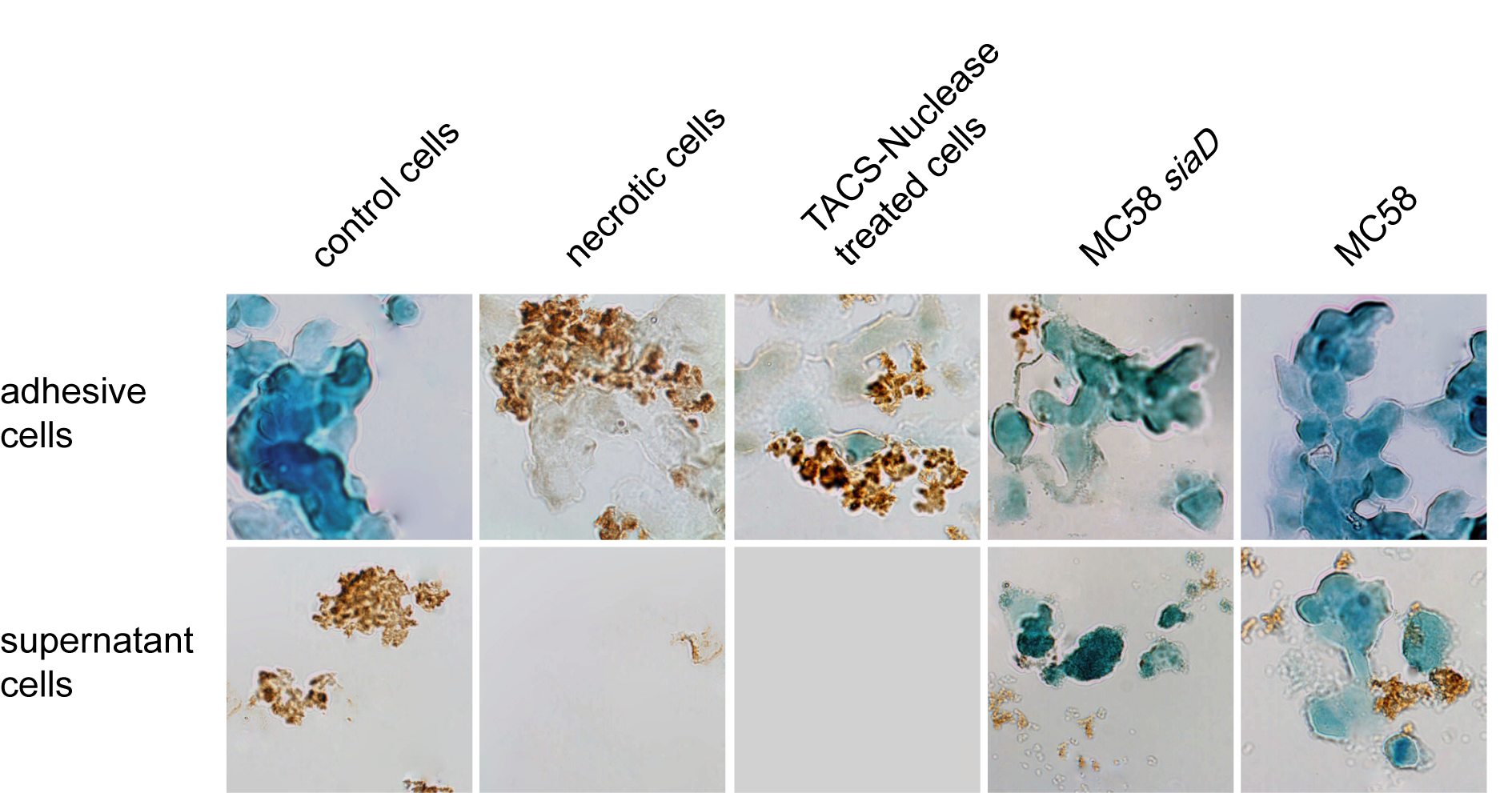

Supplement: Figure S4 — Detection of apoptosis by TUNEL staining. HBMEC were cultured on cover slides and infected with N. meningitidis MC58 and MC58 siaD for a 24 h time-period. Positive terminal deoxynucleotidyl transferase-mediated dUTP nick end labeling (TUNEL) staining was observed in cells collected from the supernatant (‘floating’ cells), while adherent cell showed rare positive apoptotic cells. Shown are representative photomicrographs from three independent experiments. (2.42 MB TIF) [file ppat.1000874.s005.tif]
