# Supplementary material for: Neisseria meningitidis Induces Brain Microvascular Endothelial Cell Detachment from the Matrix and Cleavage of Occludin: A Role for MMP-8
Source: PLoS Pathog. 2010 Apr 29;6(4):e1000874. doi: 10.1371/journal.ppat.1000874 (PMC2861698; doi:10.1371/journal.ppat.1000874)
Supplement: Table S1 — Evaluation of the rate of apoptosis in HBMEC after infection with N. meningitidis. Confluent HBMEC monolayers were infected with different MOIs of N. meningitidis MC58 and MC58 siaD. After 24 h of incubation adherent and floating cells were separately collected, washed and stained with PI-Annexin V and analyzed by flow cytometry. As a positive control for apoptosis, cells were treated with 1 µM and 5 µM Staurosporine (STS). Data present the mean ± SD of three independent experiments done in duplicate. (0.03 MB DOC) [file ppat.1000874.s006.doc]

**Table S1**

**Evaluation of the rate of apoptosis in HBMEC after infection with *N. meningitidis***

|  |  | MC58 *siaD* (MOI)  adherent cells | | MC58 *siaD* (MOI)  floating cells | | MC58 (MOI)  adherent cells | | MC58 (MOI)  floating cells | | STS | |
| --- | --- | --- | --- | --- | --- | --- | --- | --- | --- | --- | --- |
|  | control | 30 | 100 | 30 | 100 | 30 | 100 | 30 | 100 | 1 µM | 5 µM |
| Rate of apoptosis (% ± SD) | 2.3 ± 0.1 | 4.0 ± 0.9 | 5.3 ± 0.9 | 10.0 ± 2.3 | 10.8 ± 4.1 | 6.5 ± 3.1 | 5.7 ± 1.5 | 54.4 ± 8.6 | 49.5 ± 14.3 | 13.1 ± 10.7 | 59.2 ± 3.5 |
